# CAP1 (Cyclase-Associated Protein 1) Exerts Distinct Functions in the Proliferation and Metastatic Potential of Breast Cancer Cells Mediated by ERK

**DOI:** 10.1038/srep25933

**Published:** 2016-05-13

**Authors:** Haitao Zhang, Guo-Lei Zhou

**Affiliations:** 1 Department of Biological Sciences, Arkansas State University, State University, AR 72467, USA; 2 Molecular Biosciences Program, Arkansas State University, State University, AR 72467, USA

## Abstract

The actin-regulating protein CAP1 is implicated in the invasiveness of human cancers. However, the exact role remains elusive and controversial given lines of conflicting evidence. Moreover, a potential role in the proliferative transformation has largely been overlooked. Further establishing the role and dissecting underlying mechanisms are imperative before targeting CAP1 can become a possibility for cancer treatment. Here we report our findings that CAP1 exerts cell type-dependent functions in the invasiveness of breast cancer cells. Depletion of CAP1 in the metastatic MDA-MB-231 and BT-549 cancer cells stimulated the metastatic potential while it actually inhibited it in the non-metastatic MCF-7 cancer cells or in normal cells. Moreover, we demonstrate functions for CAP1 in cancer cell proliferation and anchorage-independent growth, again in a cell context-dependent manner. Importantly, we identify pivotal roles for the ERK-centered signaling in mediating both CAP1 functions. Phosphor mutants of CAP1 at the S307/S309 regulatory site had compromised rescue effects for both the invasiveness and proliferation in CAP1-knockdown cells, suggesting that CAP1 likely mediates upstream cell signals to control both functions. These novel mechanistic insights may ultimately open up avenues for strategies targeting CAP1 in the treatment of breast cancer, tailored for specific types of the highly diverse disease.

Uncontrolled cell proliferation and elevated invasiveness are the two arguably most prominent hallmarks of cancer cells^1^. Cancer metastasis, resulted from elevated invasiveness of cancer cells, accounts for the majority of cancer patient deaths. This is especially true in breast cancer, which will rarely be fatal as far as the disease does not spread to other parts of the body. On the other hand, elevated cell proliferation underlies cancerous transformation of cancer cells, which also contributes to the acquired resistance to cancer treatment such as chemotherapy.

Dynamic rearrangement of the actin cytoskeleton provides essential driving force for directional cell movement. Actin filament rich structures, such as filopodia and lamellipodia, help establish cell polarity and pull the cell forward during direction cell movement. In cancer cells, an aberrant actin cytoskeleton underlies the elevated cell motility and invasiveness. CAP (Cyclase-Associated Protein) is a versatile actin-regulating protein conserved in all eukaryotes^2^. Mammals have two CAP isoforms, CAP1 and CAP2, and CAP1 is ubiquitously expressed^3^,^4^. Recent studies, including those from our group, have established roles for mammalian CAP1 in regulating the actin cytoskeleton and cell migration^5^,^6^,^7^. Knockdown of CAP1 in mammalian cells causes actin cytoskeletal alterations that suggest reduced actin filament turnover, which is consistent with the cellular function of CAP1. As expected, depletion of CAP1 also led to reduced motility in some cells^5^,^7^. However, depletion of CAP1 in HeLa cells actually stimulated migration and invasion substantially^6^, where activation of cell adhesion signaling apparently played a key role and overcame the negative effect from the reduced actin dynamics. We identified a novel role for CAP1 in cell adhesion, by showing interactions of CAP1 with key adhesion regulators including FAK (Focal Adhesion Kinase) and Talin^6^. Cell adhesion, through integrin activation, also plays an important role in cell migration as it provides traction force essential for cell to move forward^7^. While activation of integrins can be regulated bi-directionally, in this case, intracellular stimuli cause extracellular changes in adhesion, through a so called inside-out signaling^7^,^8^. Mounting evidence over the last decade suggests involvement of CAP1 in the invasiveness of a growing list of human cancers, including pancreatic, lung and breast cancer^2^,^7^,^9^,^10^,^11^. While most of the studies so far suggest up-regulation of CAP1 in cancer and a stimulatory role in cancer invasiveness, a few lines of conflicting evidence are also available against this scenario, as elaborated below. It is therefore critical to further establish the role for CAP1 in human cancers, including that across distinct sub-types of cancer. The latter is especially important for a disease as diverse as breast cancer-in its histology, genetic lesions, proliferation, response to treatment, and propensity to metastasize^12^,^13^.

The first line of conflicting evidence is that it does not always hold true that CAP1 is pro-migratory in cells; we found depletion of CAP1 in HeLa cells actually stimulated cell motility and invasion, with the specificities verified using a rescue strategy^6^. Secondly, a protein atlas database generated from comparison of gene expression in normal tissues and human cancers at both protein and RNA levels (http://www.proteinatlas.org/ENSG00000131236-CAP1/cancer) shows that colorectal cancer had the highest percentage of cancer samples (over 50%) showing strong CAP1 staining, whereas in other cancer types percentage of cancer tissues with strong CAP1 staining was much reduced. In breast cancer, ~10% of tested cancer samples had strong CAP1 staining. It is also noted that in studies so far, a normal cell line has not always been available or included as a control for direct comparison to that of cancer cells in Western blotting. Finally, we recently identified the very first regulatory mechanism for CAP1, through phosphorylation at the S307/S309 tandem site^14^. Therefore, at least an additional dimension of regulation exists for CAP1, and regulation through CAP1 expression may not be as important for the role of the protein in the cancerous transformation and progression.

We report here our findings of cell context-dependent functions for CAP1 in the metastatic potential in breast cancer cells. We further identify a role for CAP1 in the proliferative transformation of cancer cells. Importantly, the ERK-centered signaling plays pivotal roles in mediating both CAP1 functions. In metastatic breast cancer cells, depletion of CAP1 stimulated both the invasiveness and cell proliferation, while in non-metastatic MCF-7 cancer cells it actually had opposite effects. The studies unravel novel cell type-specific functions for CAP1, and the role in proliferative transformation that has largely been overlooked, along with underlying mechanisms. These mechanistic insights carry important implications in ultimately developing strategies targeting CAP1 in the treatment of breast cancer, which is a highly diverse disease.

## Results

### No evidence supporting up-regulation of CAP1 in breast cancer cells, and a dynamic regulation for the protein

**Figure 1:**
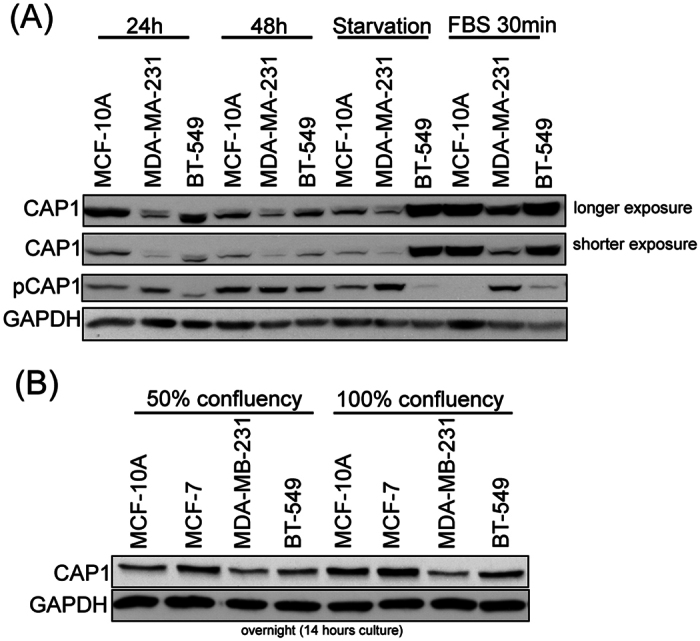
CAP1 expression and regulation in a panel of breast cancer cell lines under different culture conditions. (**A**) The effect of different culture conditions on the expression of CAP1 and phosphorylation at the S307/S309 tandem phosphor-regulatory site. Normal breast cell line MCF-10A and breast cancer cell lines MDA-MB-231 and BT-549 were cultured under normal condition for 24 hrs, 48 hrs, followed by starvation for 24 hrs or serum stimulation again for 30 minutes. Cell lysates we prepared from the cells with different treatments and were analyzed in Western blotting with the antibody against CAP1. GAPDH served as the loading control. Phosphorylation at S307/S309 was detected using the phosphor-specific antibody against CAP1 we previously developed. (**B**) The effect of growth confluency on the expression of CAP1. MCF-10A, MCF-7, MDA-MB-231 and BT-549 cells were cultured overnight to reach either 50% or 100% confluency. The CAP1 protein levels were analyzed in Western blotting, with GAPDH serving as a loading control.

Given the contradictory evidence available on up-regulation of CAP1 in human cancers including breast cancer, we examined and compared CAP1 expression in a panel of breast cancer cells. Two highly metastatic breast cancer cell lines (MDA-MB-231 and BT-549), a non-metastatic cancer cell line (MCF-7) and the normal breast epithelial cell line MCF-10A, were included^15^,^16^. As shown in Fig. 1A, firstly, CAP1 levels varied depending on the duration of culture; in both MCF-10A and BT-549 cells, cells cultured for 48 hrs had noticeably reduced CAP1 levels compared to those in a 24-hr culture. Secondly, starvation and subsequent serum stimulation both caused more substantial changes in CAP1 levels, and moreover, different cell lines responded very differently to the treatments. Serum starvation significantly up-regulated CAP1 in BT-549 cells, but not in MCF-10A and MDA-MB-231 cells. In contrast, a 30-minute serum stimulation of the starved cells led to drastically increased CAP1 levels in both MCF-10A and MDA-MB-231 cells, but did not cause further up-regulation in BT-549 cells. Thirdly, under all the above culture conditions, CAP1 levels in the metastatic MDA-MB-231 cells were consistently lower than, or similar to, those in the normal cell line MCF-10A, whereas BT-549 cells mostly had comparable levels to those in MCF-10A cells. We next looked into if the confluency of the culture may influence CAP1 expression, also including the non-metastatic cancer cell line MCF-7. As shown in the Fig. 1B, cells reached full confluency had modestly up-regulated CAP1 levels in MCF-10A, MCF-7 and BT-549 cells, as compared to the same cells at 50% confluency. However, the non-metastatic MCF-7 cells consistently had the highest CAP1 levels while the MDA-MB-231 cells had the lowest CAP1 levels. Taken together, our above results neither support up-regulation of CAP1 in breast cancer cells, nor any correlation between CAP1 expression levels and the grade of invasiveness of a cancer cell type. Thus, our findings are consistent with the data in the aforementioned database, in that CAP1 is not significantly up-regulated in breast cancer.

We also examined expression of CAP2, the other CAP isoform, in these cells to confirm that CAP1 is indeed the predominate isoform in breast cancer cells. Using a polyclonal antibody we developed against a shared sequence on human and mouse CAP2 (SEKIQEIQTFRERNR; a.a. 111–125), we found that CAP2 was virtually undetectable in breast cancer cells, whereas it was highly expressed in HeLa cells (Fig. S1). These results are consistent with limited CAP2 expression to a few specific tissue types^3^, and validate our focus on CAP1 in this study.

### CAP1 depletion stimulates invasiveness in metastatic cancer cells, but inhibits the motility in non-metastatic cancer cells

**Figure 2:**
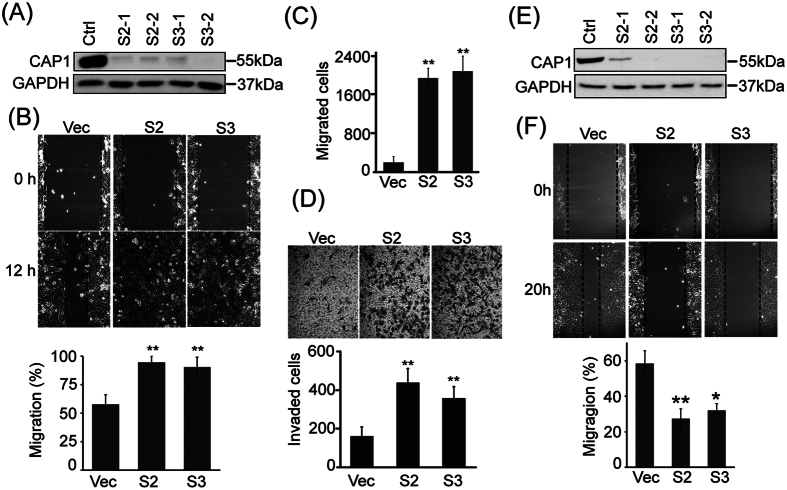
CAP1 knockdown resulted in opposing effects on the invasiveness in the metastatic BT-549 and non-metastatic MCF-7 breast cancer cells. (**A,E**) Efficient knockdown of CAP1 in the BT-549 (**A**) and MCF-7 (**E**) cancer cells was confirmed by Western blot. For each of the independent S2 and S3 shRNA constructs, two stable clones were established and used for evaluation. GAPDH serves as a loading control. (**B,F**) Wound healing assays assessing effects of CAP1 depletion on the migration capabilities in BT-549 (**B**) and MCF-7 (**F**) cells. The Y-axis in the graph represents the relative healed area and the asterisk represents S.E.M. (**P < 0.01; *P < 0.05 vs. Vec, n = 3). (**C**) Transwell migration assay results showing elevated cell migration in the CAP1 knockdown BT-459 cells. The Y-axis represents the mean of migrated cells and the asterisk represents S.E.M. (**P < 0.01 vs. Vec, n = 3). (**D**) Matrigel invasion assays showing elevated invasion capability in the CAP1 knockdown BT-549 cells. The Y-axis represents the mean of invaded cells and the asterisk represents S.E.M. (**P < 0.01 vs. Vec, n = 3). The upper panels show the representative microscopic images of the invaded cells.

To determine the role of CAP1 in modulating the metastatic potential of the breast cancer cells, we performed gene silencing by using vector-based shRNA constructs that are compatible with a rescue strategy^6^,^14^. Multiple stable knockdown clones were established in MDA-MB-231, BT-549 and MCF-7 cancer cells (and also the MCF-10A normal cells; not shown), derived from both the S2 and S3 shRNA constructs (Figs 2A,E and S2A).

**Figure 3:**
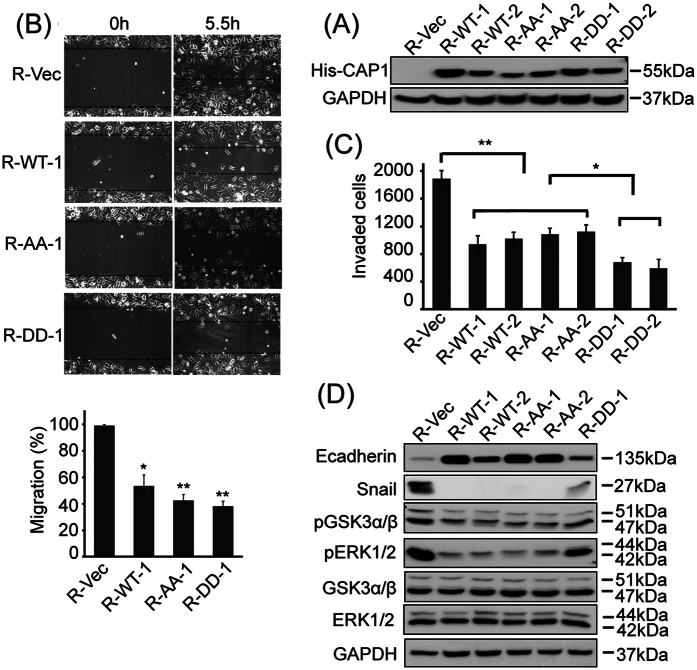
Rescue of the cell migration and invasion phenotypes and relevant regulators by the re-expression of CAP1 or its phosphor mutants in CAP1 knockdown BT-549 cells. (**A**) Western blot with anti-6xHis antibody confirmed re-expression of the exogenous WT CAP1 and the AA and DD mutants in multiples stable clones, with GAPDH serving as a loading control. (**B**) Wound healing assays showed both wild type and mutated mouse CAP1 rescued the phenotype of elevated cell migration, while expression of the AA and DD mutants further compromised cell migration. (**C**) Matrigel invasion assays showed that both wild type and mutated mouse CAP1s rescued cell invasion, but expression of the DD mutant further inhibited invasion of the CAP1 knockdown BT-549 cells. (**D**) Rescue of the expression levels of Snail and E-cadherin, as well as the activities of ERK1/2 and GSK3α/β by the re-expression of wild type CAP1 and the AA and DD mutants. Re-expression of WT CAP1 or the AA mutant efficiently rescued the alterations from CAP1 knockdown in the BT-549 cells, while the DD mutant was able to partially rescue the expression of Snail and E-cadherin, or the activities of ERK1/2 and GSK3α/β. The experiments were repeated at least three times with similar results.

We next tested effect of CAP1 depletion on migration of cancer cells, by conducting both wound healing and Transwell migration assays. CAP1 depletion substantially stimulated the migration capability in both the BT-549 (Fig. 2B,C) and MDA-MB-231 metastatic cancer cells (Fig. S2B,C), which is very similar to the case in HeLa cells^6^. In contrast, CAP1 knockdown in the MCF-7 cells greatly reduced cell migration instead (Fig. 2F), which is consistent with the phenotypes observed in most cells so far, including normal cells and pancreatic cancer cells^5^,^10^. We further tested effect of CAP1 depletion on Matrigel invasion in BT-549 and MDA-MB-231 cells, which mimics tissue invasion in the body, and found that consistent with the migration phenotypes, CAP1 depletion also remarkably stimulated Matrigel invasion in these cells (Figs 2D and S2D). Furthermore, to confirm that these effects were truly derived from depletion of CAP1, we re-expressed CAP1 in the CAP1-depleted BT-549 cells, using an expression plasmid harboring introduced mismatches within the shRNA target sequences to avoid recognition and cleavage of the mRNA by the shRNA present in the stable cells (in a way without altering the amino acid sequence coded). Re-expression of the 6xHis-Xpress tagged CAP1 in these cells was confirmed in Western blotting (Fig. 3A; the two lanes next to the vector control), and the re-expression indeed rescued (reduced) motility in the CAP1-knockdown cells (Fig. 3B). Moreover, re-expression of CAP1 also rescued the elevated Matrigel invasion in the knockdown cells (Fig. 3C). These results support that the stimulatory effect of CAP1 depletion on the invasiveness of metastatic cancer cells were truly derived from CAP1 knockdown. Thus, depletion of CAP1 caused opposing effects in the metastatic and non-metastatic breast cancer cells on cell migration and invasion.

### CAP1 depletion leads to distinct alterations in the pro-migratory actin cytoskeletal structures

Protrusive and invasive subcellular structures help the cell navigate through the ECM (Extracellular Matrix) and into the vasculature. Lamellipodia and filopodia are such protrusive filament actin-based subcellular structures important for cancer cell migration and invasion. We tested how CAP1 depletion may have impacted these actin cytoskeletal structures in the different breast cancer cells.

**Figure 4:**
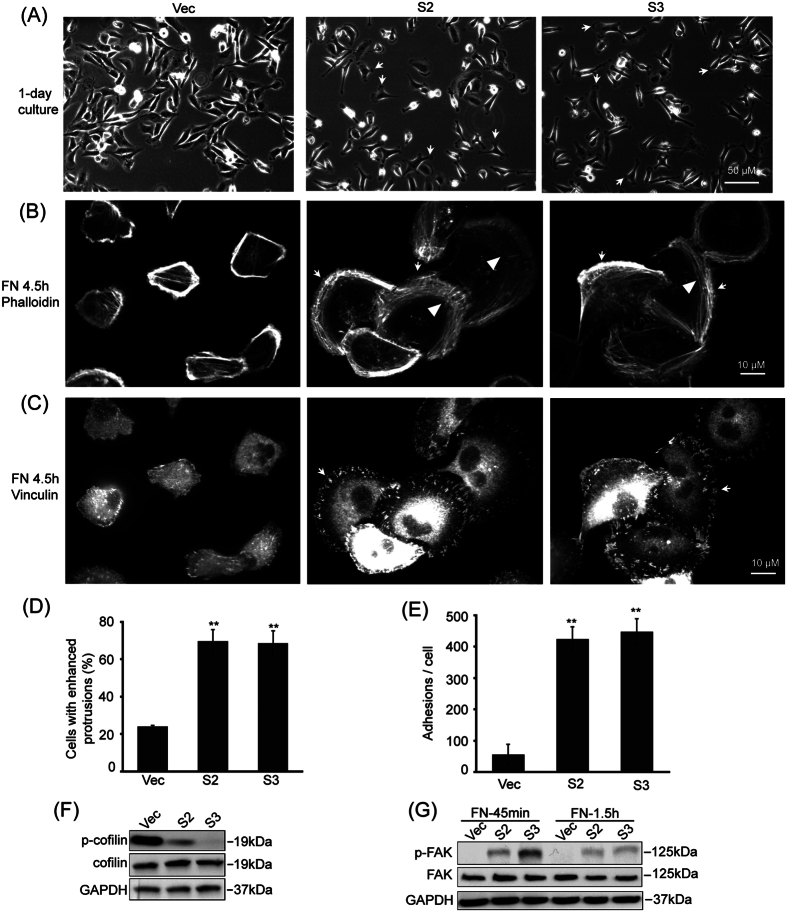
CAP1 knockdown promoted the formation of migratory subcellular structures in the metastatic MDA-MB-231 cancer cells. (**A**) Bright field images of control (Vec) and stable knockdown cells (S2 and S3) show cell morphological changes. The arrows indicated the enhanced protrusions in the knockdown cells. (**B**) Confocal images showing the actin cytoskeleton stained with fluorescent Phalloidin in cells cultured on fibronectin-coated surface for 4.5 hrs. Arrows indicate the lamellipodia and the arrowheads indicate the actin arcs at the leading edges. (**C**) Confocal images showing focal adhesions by stained with a vinculin antibody. Arrows indicate the focal adhesions at the leading edge of cells cultured on fibronectin-coated Mattek dishes for 4.5 hrs. (**D**) Statistic analysis of cells with enhanced protrusions in the control and CAP1 knockdowns cells in (**A**). The total cell number (at least 70) and number of cells with enhanced protrusions per field were counted in five random fields. The percentage of cells with enhanced protrusions was calculated, analyzed in Student’s *t*-test and shown in the graph with the error bar representing S.D. (**P < 0.01 vs. Vec, n = 5). (**E**) Statistic analysis of the number of focal adhesions per cell. The numbers of focal adhesions per cell (**C**) were counted using the Image J program, and 25 cells per filed were counted. The experiment was repeated three times and the average numbers were represented in the graph with the error bars representing S.D. (**P < 0.01 vs. Vec, n = 3). (**F**) Knockdown of CAP1 led to activation of cofilin in MDA-MB-231 cells. Phosphorylation at Ser3 on cofilin was detected and compared between control and CAP1 knockdown cells. (**G**) Knockdown of CAP1 in MDA-MB-231 cells caused activation of FAK. Phosphorylation at Y397 on FAK was detected and compared between the control (Vec) and CAP1 knockdown cells.

**Figure 5:**
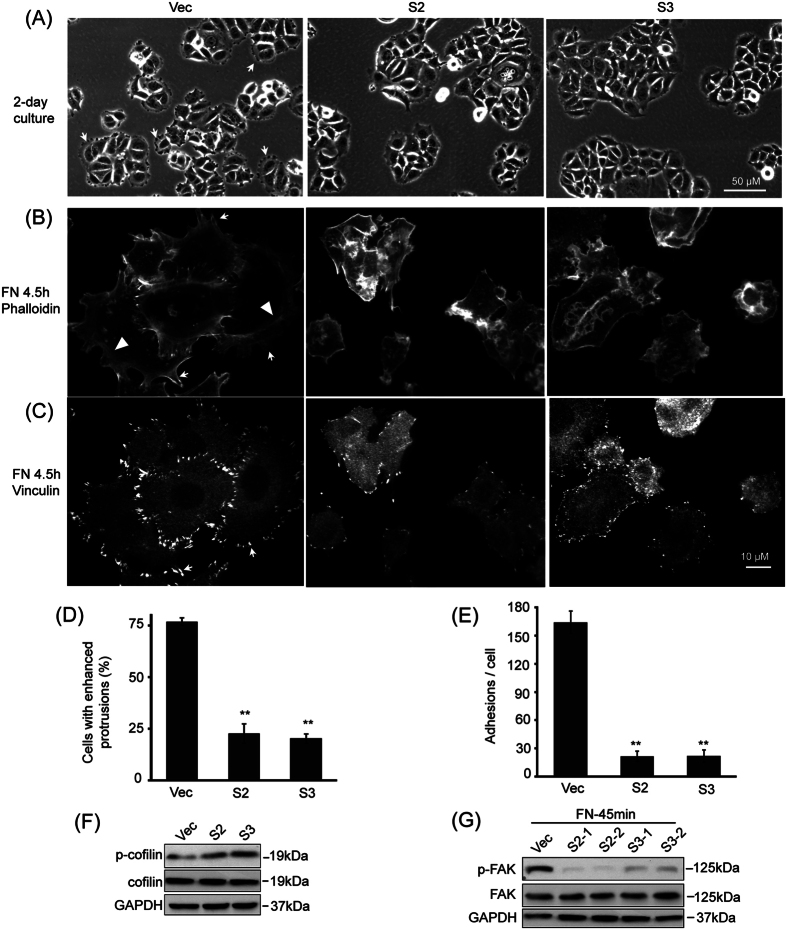
CAP1 knockdown in the non-metastatic MCF-7 cells inhibited the formation of the migratory subcellular structures. (**A**) Bright field images of control (Vec) and stable knockdown cells (S2 and S3) show cell morphological alterations associated with migration capability. The arrows indicate the protrusions developed at the leading edge of the control cells. (**B**) Confocal images showing the actin cytoskeleton stained with fluorescent Phalloidin. Arrows indicate the protrusions and the arrowheads indicate the actin arcs at the leading edge. (**C**) Confocal images showing the focal adhesions stained with a vinculin antibody. Arrows indicate the focal adhesions at the leading edge of cells 4.5 hours after seeded onto fibronectin-coated Mattek dishes. (**D**) Statistic analysis of cells with enhanced protrusions in the control and CAP1 knockdowns cells in (**A**). The total cell number (at least 70) and number of cells with enhanced protrusions per field were counted in five random fields. The percentage of cells with enhanced protrusions was calculated, analyzed in Student’s *t*-test and shown in the graph with the error bar representing S.D. (**P < 0.01 vs. Vec, n = 5). (**E**) Statistic analysis of the number of focal adhesions per cell. The numbers of focal adhesions per cell (**C**) were counted using the Image J program, and 25 cells per filed were counted. The experiment was repeated three times and the average numbers were represented in the graph with the error bars representing S.D. (**P < 0.01 vs. Vec, n = 3). (**F**) Knockdown of CAP1 led to activation of cofilin in MCF-7 cells. Phosphorylation at Ser3 on cofilin was detected and compared between control and CAP1 knockdown cells. (**G**) Knockdown of CAP1 in MCF-7 cells caused inactivation of FAK. Phosphorylation at Y397 on FAK was detected and compared between the control (Vec) and CAP1 knockdown cells.

Bright field imaging revealed remarkable morphological changes in CAP1-depleted MDA-MB-231 (Fig. 4A) and BT-549 cancer cells (Fig. S3A). CAP1 knockdown MDA-MB-231 cells became more rounded and many cells had developed broad lamellipodia under both normal culture (Fig. 4A,D; indicated with arrows) and on fibronectin-coated surface (Fig. 4B; indicated with arrows). The changes in cell morphology and development of broad lamellipodia were even more remarkable in the BT-549 cells (Fig. S3A,B,D), where most cells had a rounded-up morphology and harbored robust lamellipodia. These phenotypes are consistent with the elevated migration and invasion (Figs 2A–D and S2). In contrast, depletion of CAP1 significantly inhibited formation of lamellipodia in the non-metastatic MCF-7 cells (Fig. 5A,B,D). Over 75% of the control cells (at the peripheral of cell clusters, MCF-7 cells tend to form clusters in culture) harbored protrusions as indicated by the arrows, whereas the CAP1 knockdown MCF-7 cells had substantially diminished protrusions. We also confirmed that re-expression of CAP1 in the knockdown BT-549 cells rescued the phenotype of enhanced lamellipodia, as the cells re-expressing CAP1 were not as rounded up (Fig. S4A; the two top panels). Lamellipodia formation in the BT-549 cells cultured on fibronectin-coated surface had a similar result; the knockdown cells were already well spread and harbored broad lamellipodia at the leading edge 45 minutes after plated on coated surface; in contrast, many of the rescue cells did not harbor broad lamellipodia but instead had a star-fish like morphology (Fig. S4B; the two top panels).

We observed further alterations in another pro-migratory cytoskeletal structure, called lamella, in the knockdown metastatic cells. Migrating cells advance by net protrusions at the leading edges and retract at the trailing edges^17^. Two regions that define the leading edge are the lamellipodia, extending ~3–5 μm from the cell front edge, and the lamella found immediately behind the lamellipodium composed of actin arcs in association with focal adhesions^18^,^19^. The actin arcs serve as a structural element underlying the temporal and spatial connection between the leading edges and the cell bodies during cell movement^20^. Confocal microscopy revealed highly polarized actin cytoskeleton structures in the CAP1 knockdown MDA-MB-231 and BT-549 cells, with enhanced actin arcs in the lamella area (Figs 4B and S3B; indicated with arrowheads). In contrast, control cells did not develop these structures and were poorly polarized. Interestingly, effect of CAP1 depletion was again completely opposite in the MCF-7 cells (Fig. 5B) in this regard: while in control MCF-7 cells serrated filopodia formed at the leading edges (indicated with arrows) followed by the actin arcs in the lamella region (indicated with arrowheads), these structures were not observed in the CAP1 knockdown cells. Therefore, the distinct actin cytoskeletal alterations in metastatic and non-metastatic breast cancer cells are consistent with the cancer cell migration and invasion phenotypes. Finally, we also looked into potential alterations in the activity of the key ADF (Actin-Deploymerization Factor) family member cofilin, which is central to actin dynamics, in CAP1 knockdown cells. Interestingly, the cofilin activity is significantly elevated in the CAP1 knockdown MDA-MB-231 cells, as indicated by reduced phosphorylation at Ser3 (Fig. 4F), which is again similar to the case in HeLa cells and consistent with increased invasiveness of these cells. In contrast, in MCF-7 cells, knockdown of CAP1 actually led to modest inactivation of cofilin, as indicated by the slightly elevated Ser3 phosphorylation (Fig. 5F).

### CAP1 depletion in breast cancer cells causes distinct alterations in focal adhesions

We first identified a role for CAP1 in regulating FAK and focal adhesions in HeLa cells^6^. Focal adhesions couple the cell membrane to the actin cytoskeleton and the ECM, and serve as traction sites for generating the traction force necessary to pull the cell body forward during migration^21^. We looked into potential changes in the focal adhesions in the CAP1 knockdown breast cancer cells. As shown in the confocal images of vinculin-stained cells, a large number of bright focal adhesion dots were observed at the peripheral (predominantly at the leading edge) of the CAP1-depleted MDA-MB-231 (Fig. 4C,E). In contrast, focal adhesions in the control metastatic cancer cells were much less well developed (Fig. 4C,E). CAP1 knockdown in BT-549 cells showed similar phenotypes (Fig. S3C,E). These phenotypes are very similar to that in HeLa cells^6^, and we confirmed that FAK was indeed activated in CAP1 knockdown MDA-MB-231 cells as well (Fig. 4G). Opposing to this phenotype, CAP1 knockdown MCF-7 cells had much reduced focal adhesions than control cells (Fig. 5C,E), and consistently the knockdown cells also had significantly reduced FAK activity (Fig. 5G). Since cell adhesion is crucial for cell spreading, the cells with enhanced focal adhesions (knockdown MDA-MB-231 cells and the control MCF-7 cells) consistently had significantly larger cell size, especially at early stage (4.5 hours) after cells were plated onto fibronectin-coated surface (Figs 4A–C,5A–C and S5A,B).

We further looked into effect of CAP1 depletion on cell spreading on the fibronectin-coated surface to assess effects on cell-ECM interaction. CAP1 knockdown MDA-MB-231 cells (Fig. S5A,C) displayed much enhanced cell-ECM interaction compared to the control cells, as indicated by the full spreading of most cells 45 minutes after cells were plated. Moreover, the spreading itself was not as complete in the control cells either. In contrast, CAP1 knockdown in the MCF-7 cells inhibited this interaction; more than 70% of the control MCF-7 cells were fully spread with developed lamellipodia 90 minutes after cells were plated, while only less than 30% of the CAP1 knockdown cells were spread and even in these cells many did not have a full spreading (Fig. S5B,D).

In summary, alterations in the actin cytoskeleton, cancer cell adhesion and invasiveness consistently support cell context-dependent functions for CAP1 in regulating cancer cell invasiveness in the metastatic and non-metastatic breast cancer cells.

### The ERK-GSK3/Snail/E-cadherin axis mediates CAP1 function in the invasiveness of breast cancer cells

**Figure 6:**
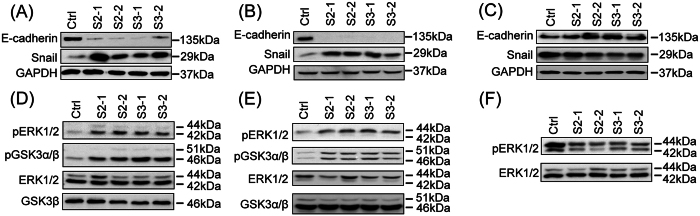
Distinct effect of CAP1 knockdown on the ERK-GSK3/Snail/E-cadherin axis in the metastatic and non-metastatic breast cancer cells. (**A,B**) CAP1 knockdown caused up-regulation of Snail, a transcription suppressor of E-cadherin, and consistently down-regulated E-cadherin in the BT-549 cells (**A**) and MDA-MB-231 cells (**B**). GAPDH served as a loading control. (**C**) CAP1 knockdown led to down-regulation of Snail, and consistently up-regulation of E-cadherin in the MCF-7 cells. GAPDH served as a loading control. (**D,E**) CAP1 knockdown led to activation (elevated phosphorylation at Thr202/Tyr204) of ERK1/2 as well as inactivation of GSK3α/β (elevated phosphorylation at S21/S9) in both the BT-549 (**D**) and MDA-MB-231 cells (**E**). Total ERK and total GSK3β served as loading controls. (**F**) CAP1 knockdown inactivated ERK1/2 in MCF-7 cells, as indicated by the reduced phosphorylation at Thr202/Tyr204) in the CAP1 knockdown cells. Total ERK served as a loading control. All experiments were repeated at least three times with similar results.

Elevated invasiveness of tumor cells is reminiscent of EMT (Epithelial-Mesenchymal Transition), the dedifferentiation of epithelial cells toward a mesenchymal state that is believed to be a key step toward cancer metastasis^22^,^23^. Loss of E-cadherin, a protein in the cell-cell junction, is a hallmark of EMT in cancer cells since they lose connections to each other. We found that CAP1 depletion indeed reduced the expression of E-cadherin in BT-549 and MDA-MB-231 cancer cells (Fig. 6A,B), consistent with the elevated invasiveness caused by CAP1 depletion. In contrast, CAP1 knockdown in the MCF-7 cells actually led to up-regulation of E-cadherin (Fig. 6C), suggesting reduced EMT in CAP1 knockdown MCF-7 cells. Importantly, re-expression of CAP1 in the CAP1-depleted BT-549 cells again rescued the E-cadherin levels; both of the clones tested re-expressing Wild Type (WT) CAP1 (R-WT-1 and R-WT-2) had significantly higher E-cadherin levels as compared to that in CAP1 knockdown cells (Fig. 3D).

Several transcription factors have been implicated in controlling EMT, such as Snail/Slug, Twist and ZEB1^24^. Snail plays a fundamental role in breast cancer metastasis by repressing E-cadherin expression and inducing EMT in breast cancer cells^25^,^26^,^27^,^28^. To test if Snail may mediate the regulation of E-cadherin by CAP1, we looked into Snail expression and found that CAP1 knockdown BT-549 and MDA-MB-231 cancer cells had significantly up-regulated Snail levels (Fig. 6A,B). In contrast, in the CAP1 knockdown MCF-7 cells, Snail was moderately down-regulated instead (Fig. 6C). Furthermore, re-expression of CAP1 in the CAP1-depleted BT-549 cells again rescued the Snail level (Fig. 3D). These results strongly support that Snail mediates the CAP1 function in regulating E-cadherin levels and EMT.

We next dissected signaling molecules that may function between CAP1 and Snail. Snail is highly unstable and regulated at both transcriptional and post-translational levels. Snail transcription can be activated by the ERK1/2 (MAPK)-Elk1 axis^29^,^30^, while Snail expression is inhibited by GSK3 (Glycogen Synthase Kinase 3)^31^ as phosphorylation of Snail by GSK3 promotes degradation of Snail^32^. We thus examined the alterations in the activities of ERK1/2 (Extracellular signal-Regulated Kinase) and GSK3. We indeed found that the activity of ERK1/2 was elevated in CAP1 knockdown MDA-MB-231 and BT-549 cells, as indicated by the increased phosphorylation at Thr202/Tyr204 (Fig. 6D,E). Interestingly, the activity of GSK3 was simultaneously inhibited, as indicated by the elevated inhibitory phosphorylation at Ser21/Ser9 of GSK3α/β (Fig. 6D,E). Opposing to the case in the metastatic cancer cells, CAP1 knockdown in the MCF-7 cells reduced the activity of ERK1/2 instead (Fig. 6F), whereas no significant changes in GSK3 activity was observed (not shown). Importantly, re-expression of CAP1 in the CAP1 knockdown BT-549 cells also rescued the activities of ERK and GSK3 (Fig. 3D). Taken together, these results support that the ERK-GSK3/Snail/E-cadherin axis mediates the functions of CAP1 in cancer cell invasiveness and EMT.

### Cell context-dependent functions for CAP1 in cancer cell proliferation and anchorage-independent growth

**Figure 7:**
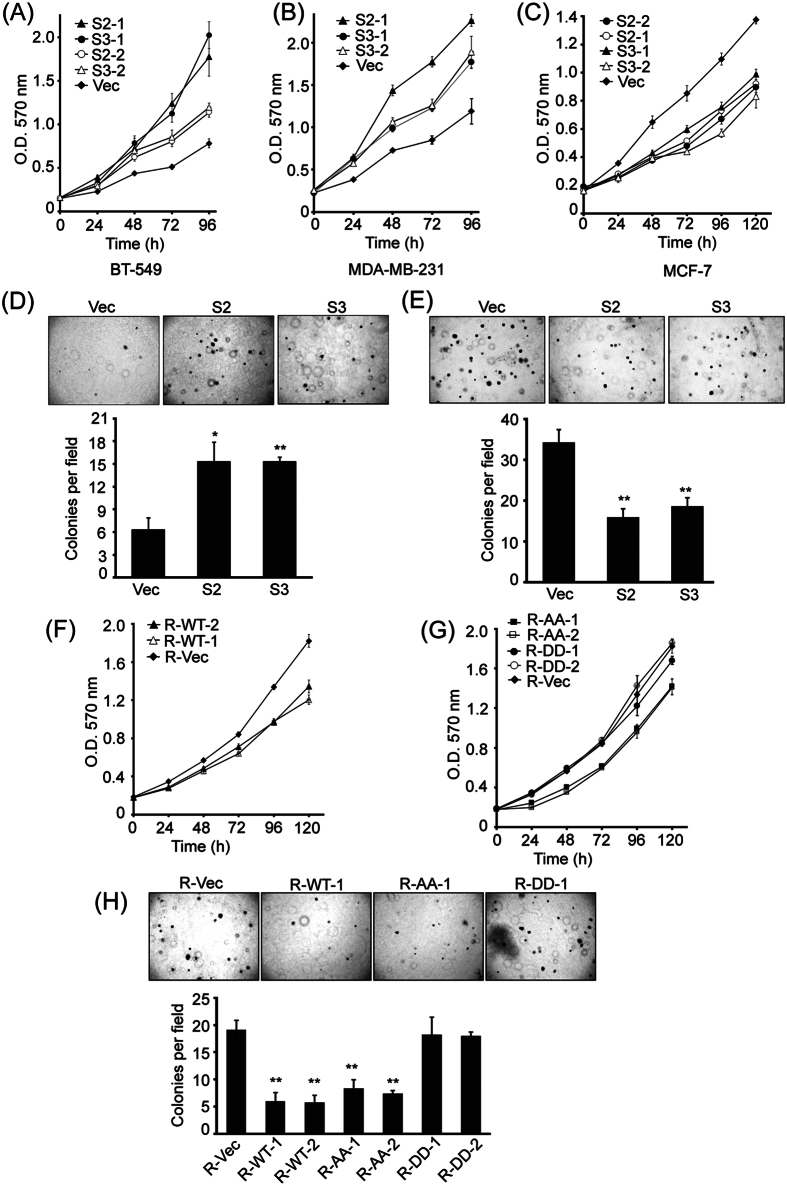
Roles for CAP1 in the proliferation and anchorage-independent growth of breast cancer cells. (**A,B**) MTT assays revealed that CAP1 knockdown promoted the proliferation of the metastatic BT-549 (**A**) and MDA-MB-231 (**B**) breast cancer cells. (**C**) MTT assays showed that CAP1 knockdown inhibited the proliferation of MCF-7 cells. **(D,E**) Effects of CAP1 knockdown on colony formation on soft agar. Representative micrographs (upper panel) and quantified results (lower panel) of the crystal violet stained cell colonies of BT-549 (**D**) and MCF-7 (**E**) cells. (*P < 0.05; **P < 0.01 vs. Vec, n = 2). Results in each graph are based on two independent experiments, with the error bar representing S.D. (**F**) MTT assays showed that re-expression of WT CAP1 in the CAP1 knockdown BT-549 cells rescued the elevated cell proliferation from depletion of CAP1. (**G**) MTT assays showed that re-expression of AA mutant in the CAP1 knockdown BT-549 cells rescued the elevated cell proliferation from depletion of CAP1, while the DD mutant was unable to rescue the phenotype as efficiently. All of the MTT experiments were repeated three times, with the error bar representing S.D. (**H**) The AA mutant rescued the phenotype of increased colony formation in the CAP1 knockdown cells more efficiently than the DD mutants. Representative micrographs (upper panel) and quantified results (lower panel) of the crystal violet stained cell colonies of BT-549 cells. (*P < 0.05; **P < 0.01 vs. Vec, n = 2).

Since CAP1 depletion led to altered activity of ERK, a key regulator of cell proliferation, we speculated that CAP1 may also regulate proliferative transformation in breast cancer cells. To this end, we performed MTT and soft agar colony formation assays to test the potential effects of CAP1 depletion on cell proliferation and anchorage-independent growth. Growth of cancer cells on semi-solid soft agar surface to form colonies is considered the most stringent *in vitro* assay for detecting malignant transformation of cells. Depletion of CAP1 in both BT-549 and MDA-MB-231 cells, consistent with the activation of ERK in these cells, indeed stimulated cell proliferation (Fig. 7A,B). In the MCF-7 cells; however, CAP1 knockdown actually inhibited cell proliferation (Fig. 7C), consistent with reduced ERK activity. We further found that CAP1 knockdown promoted the colony formation on soft agar in BT-549 cells (Fig. 7D), yet it again had an opposite effect in MCF-7 cells by inhibiting the colony formation (Fig. 7E). We next tested CAP1-rescued BT-549 cells in the MTT and soft agar assays, and found that re-expression of CAP1 also rescued the phenotypes detected in MTT (Fig. 7F) and soft agar assays (Fig. 7H). Therefore, similar to the opposite effects of CAP1 depletion on the invasiveness cancer cells, knockdown of CAP1 also had opposite effects on the proliferation of metastatic and non-metastatic cells, consistent with the opposing alterations of ERK activity. Since potential alterations in cell apoptosis may also underlie changes detected in the cell proliferation and colony formation assays, we looked into Bcl-2 expression and also conducted Hoechst staining^33^; however, no significant alterations were observed in the knockdown cells (not shown). Thus our results support that altered ERK activity, but not cancer cell apoptosis, was responsible for the effects of CAP1 knockdown on cell proliferation and anchorage-independent colony formation in breast cancer cells.

### Roles for S307/S309 phosphorylation in CAP1 functions in regulating both proliferation and invasiveness of cancer cells

Interestingly, phosphorylation at the S307/S309 tandem regulatory site undergoes a dynamic regulation in cancer cells responding to serum starvation and stimulation (Fig. 1A). These results suggest that cell signals may control both the invasiveness and proliferation of breast cancer cells by regulating CAP1 function through phosphorylation. To test this, we looked into rescue effects by two phosphor mutants in the CAP1-depleted BT-549 cells: the AA mutant mimics unphosphorylatable S307/S309 while the DD mutant mimics phosphorylated residues. Stable clones re-expressing each mutant were established and verified by Western blotting (Fig. 3A). When compared to the control cells harboring an empty vector (R-Vec), both the AA and DD mutants rescued the phenotypes in cell migration (Fig. 3B) and also in Matrigel invasion (Fig. 3C). However, the AA and DD mutants actually further reduced cell motility as compared to WTCAP1 (Wild Type CAP1; Fig. 3B). This is somewhat confusing, since phosphor mutants that cannot undergo reversible phosphor-regulation are expected to have compromised functions^14^; accordingly, motility of the mutant cells is expected to be higher than the cells rescued by WTCAP1. When we examined cell morphologies, we found that while both the AA and DD mutants partially rescued the cell morphology compared to WTCAP1 (R-WT), suggesting that the mutants are partially functional in that regard, the mutant cells did not spread as well as the cells re-expressing WTCAP1 in overnight culture (Fig. S4A). Furthermore, unlike the cells re-expressing WTCAP1, the mutant cells shortly after being plated onto fibronectin-coated surface also developed big “lamellipodia”, but they were not precisely localized to the leading edges (Fig. S4B; indicated with arrows). This unique lamellipodia phenotype, combined with the poor spreading, may have caused problems in polarization and gaining sufficient traction force, and collectively they likely have led to the unexpectedly low rate of directional movement. We next tested if the mutants could rescue the alterations in the signaling molecules in the ERK-GSK3/Snail/E-cadherin axis. As shown in the Western blot results (Fig. 3D), the AA mutant rescued the expression of both Snail and E-cadherin similarly well as WTCAP1; however, the DD mutant only partially rescued them. This is consistent with our previous conclusion that DD appears to be the “inactive” form relative to the AA mutant^14^. We also tested effects of the AA and DD mutants on cell proliferation, and found that in both MTT proliferation and soft agar assays, the AA mutant rescued the cell proliferation phenotypes more efficiently than the DD mutant in the BT-549 cells (Fig. 7G,H). This is also consistent with the significantly compromised rescue effect of ERK activity by the DD mutant (Fig. 3D). Taken together, our results support that CAP1 also functions in breast cancer cell proliferation, again mediated by ERK-centered cell signaling. Moreover, phosphorylation at the regulatory site on CAP1 by upstream cell signals likely regulates CAP1 functions in both the proliferative transformation and the metastatic potential of breast cancer cells.

## Discussion

Consistent with its cellular function as a key actin-regulating protein, mounting evidence supports involvement of CAP1 in the invasiveness of human cancers. While majority of the evidence to date suggests a stimulatory role, conflicting evidence also exists against up-regulation of CAP1 in human cancers and a universal role in promoting cancer invasiveness. Results from our well-controlled and in-depth studies do not support up-regulation of CAP1 in breast cancer cells, and further, we reveal cell context-dependent functions for CAP1 in the invasiveness, as well as proliferation of breast cancer cells, along with underlying mechanism where ERK signaling plays a pivotal role. While up-regulated CAP1 universally stimulates the invasiveness of human cancers may be a tempting model, the role for CAP1 in human cancers does not appear to be that simple and clear-cut. In line with this, there have been reports that overexpression or constitutive activation of a pro-migratory protein does not always stimulate cell motility. We found CAP1 expression undergoes a dynamic regulation, suggesting that culture conditions may have influenced the conclusions in previous studies when comparing CAP1 expression between cell lines. These novel mechanistic insights contribute to further defining and clarifying the role for CAP1 in human cancers, which is especially important for a disease as diverse as breast cancer, where thousands of genes may contribute to its pathophysiologies, and genomic and transcriptional characteristics of 51 breast cancer cell lines mirror those of 145 breast cancer tumors^16^.

We identify opposing roles for CAP1 in the invasiveness of metastatic and non-metastatic breast cancer cells. This is the first report for an inhibitory role for CAP1 in the invasiveness of human cancer cells, other than our previous report of such a role in HeLa cells^6^. Moreover, it is also novel that CAP1 actually fulfills cancer cell type-dependent functions, suggesting distinct roles for the protein in different subtypes of cancer within the same cancer. Importantly, we further identify a role for CAP1 in proliferative transformation of cancer, which has largely been overlooked due to the cellular function of CAP1. We again unravel cell context-dependent functions for CAP1, where ERK-centered signaling also mediates the function. Finally, we show evidence suggesting roles for S307/S309 phosphorylation in both CAP1 functions. Therefore, CAP1 may mediate upstream cell signals or physiological stimuli to control both the invasiveness and proliferation of cancer cells. These mechanistic insights may ultimately lead to therapeutic strategies targeting CAP1 or its peripheral cell signals in breast cancer treatment.

Knockdown of CAP1 in metastatic cancer cells stimulated migration and invasion, similar to the case in HeLa cells^6^. However, in the non-metastatic MCF-7 cancer cells and normal MCF-10A cells it actually caused opposite phenotypes, which is similar to those reported in most studies to date, ranging from fibroblasts to a few cancer types, such as pancreatic and lung cancer^9^,^10^ (we have verified that CAP1 is required for the invasiveness in pancreatic cancer cells; unpublished results from our group). Thus, CAP1 likely plays cancer type-specific, and further, cell context-dependent roles in the invasiveness of human cancers. Importantly, our improved experimental strategy allows well controlled studies: we used multiple shRNA constructs targeting independent sequences to minimize off-target risk of the RNAi approach, and we further confirmed the specificity of the phenotypes using a rescue paradigm. The invasiveness phenotypes in the CAP1 knockdown metastatic cancer cells were also supported by the phenotypes in lamellipodia, actin arcs and EMT. Similar to HeLa cells^6^, depletion of CAP1 in the metastatic cancer cells also led to remarkably enhanced focal adhesions, suggesting activation of integrin and FAK, as well as activation of cofilin. Activated FAK not only promotes the formation and turnover of focal adhesions^34^, but can also promote the formation of lamellipodia^35^,^36^. Thus, the elevated integrin/FAK activities probably stimulated the enhanced lamellipodia in the knockdown metastatic cancer cells. In contrast, CAP1 knockdown MCF-7 cells had substantially reduced focal adhesions along with reduced FAK activity. Moreover, depletion of CAP1 in the MCF-7 cells did not cause activation of cofilin either. How the depletion of CAP1 led to the opposing phenotypes in the actin cytoskeleton and focal adhesions in metastatic and non-metastatic breast cancer cells remains to be elucidated. We also tested if alterations in the 14-3-3 adaptor proteins may also account for the effects of CAP1 depletion on cancer cell invasiveness; the CAP homologues in *L.edodes* and the fission yeast interact with the 14-3-3 protein^37^, and the sigma and Zeta isoforms of the 14-3-3 protein have been implicated in the invasiveness and proliferation of breast cancer^38^,^39^. However, we did not detect any alterations in the expression or subcellular localization of the 14-3-3 isoforms in the knockdown cells (not shown).

Our studies delineate a pivotal role for the ERK-GSK3/Snail/E-cadherin axis in mediating both CAP1 functions in breast cancer cells. Repression of E-cadherin by Snail directly results in metastasis of breast cancers^27^,^28^,^40^, and the protein levels of Snail are oppositely controlled by ERK and GSK3. Indeed, depletion of CAP1 in the metastatic cancer cells stimulated the activity of ERK while simultaneously inhibited the activity of GSK3; this fits perfectly with the regulatory mechanisms leading to up-regulated Snail. The cell spreading phenotypes also suggest that CAP1 knockdown in the metastatic cancer cells promoted the interaction of transmembrane receptors with fibronectin, a major component of ECM. The activation of integrins, responsible for cell-ECM interactions, has been shown to also activate the MAPK/ERK signal^41^,^42^. Thus, activation of integrins/FAK through an inside-out signaling by CAP1 knockdown in metastatic cancer cells has most likely directly activated ERK, which may subsequently inactivate GSK3^43^. Another possible mechanism for ERK activation may be through activation of the Abl tyrosine kinase; budding yeast CAP interacts with Abl^44^ and if the interaction is conserved in mammals then depletion of CAP1 may cause altered Abl activity, which can activate ERK through Rap1 and B-Raf^45^. ERK is a well-documented regulator of cell proliferation that promotes cell cycle by up-regulating the Cyclin D1 levels^46^,^47^. The simultaneous inactivation of GSK3 may also contribute to the elevated cell proliferation, since GSK3 promotes the degradation of oncogene beta-Catenin^48^, which stimulates breast cancer cell proliferation^49^. Further efforts will be directed toward dissecting potential roles played by FAK and Abl in leading to the altered ERK activities in the CAP1 knockdown breast cancer cells.

Phenotypes in cell proliferation and anchorage-independent growth in CAP1 knockdown cells consistently support a role for the protein in cancer cell proliferation, which we show is mediated by the ERK signaling. Furthermore, the specificity of the phenotypes was confirmed by the rescue experiments in BT-549 cells. Noteworthy, our experimental paradigm allows a more appropriate platform for these studies; in addition to the rescue approach, the stable knockdown paradigm allows for studies that take longer time span. This is difficult in a transient knockdown, since the depleted target protein will typically come back within 72–96 hrs following the transfection. Considering that it takes ~24–48 hrs to reach efficient knockdown of a protein, this likely will leave too narrow a time window for reliably performing experiments such as cell proliferation assays. Identification of the role for CAP1 in cell proliferation is somewhat unexpected, since mammalian CAP1 does not appear to mediate the Ras signal like it does in yeast. However, FAK and Abl kinases, which are now linked to CAP1, both can potentially regulate ERK. We identify opposite functions for CAP1 in both the metastatic potential and proliferation of metastatic and non-metastatic breast cancer cells. We found consistent alterations in ERK activity, which most likely is caused by altered FAK/integrin activation. However, how the situation in non-metastatic cancer cells can be exactly opposite to that in the metastatic cancer cells remains to be elucidated; more knowledge on CAP1 and its interactions with other proteins and signaling pathways likely will be necessary before a more stimulating discussion can become possible. Also, proliferative and morphological transformations are often found to be interconnected, in ways such as under the regulation of the same signaling molecules or pathway; the interconnection is also consistent with the fact that biological processes are rarely isolated from each other in living systems.

In summary, we unravel cell context-dependent functions for CAP1 in the invasiveness of breast cancer cells. Moreover, we further identify a role for CAP1 in regulating proliferative transformation of cancer cells, with ERK signaling playing pivotal roles in mediating both cell functions. The findings help clarify some of the confusions surrounding the controversial roles of CAP1 in breast cancer and human cancers as a whole. Along with the mechanistic insights, identification of the cell context-dependent functions for CAP1 in breast cancer cells may help ultimately develop effective strategies targeting CAP1 in the treatment of breast cancer, that are tailored for sub-types of the highly diverse disease.

## Materials and Methods

### Cell lines and cell culture reagents

Normal MCF-10A cells and breast cancer cell lines MCF-7, MDA-MB-231 and BT-549 were obtained from ATCC. MCF10A cells were maintained in DME/F12 medium supplemented with 5% equine serum, 20 ng/mL EGF, 10 μg/mL insulin, 100 ng/mL cholera toxin and 500 ng/mL hydrocortisone. MCF-7 and MDA-MB-231 cells were maintained in DMEM supplemented with 10% fetal bovine serum. BT-549 cells were maintained in RPMI-1640 medium supplemented with 10% fetal bovine serum.

### Expression plasmids and establishment of CAP1 knockdown and re-expression stable cells

The vector-based shRNA constructs S2 (nucleotides 519–537) and S3 (nucleotides 1074–1092) that target human CAP1 have been described previously^33^. After transfection, cells were selected with 500 μg/ml G418 for two weeks. Plasmids that harbor introduced mismatches for re-expressing mouse CAP1 in the CAP1 knockdown BT-549 cells were described previously^6^,^14^. Stable re-expression clones were established through selection with 80 μg/ml Zeocin.

### Antibodies and Western blotting

The monoclonal human CAP1 antibody was previously described^50^. Antibodies against GAPDH and GSK3 beta antibodies were from Santa Cruz Biotechnology Inc. (Santa Cruz, CA). E-cadherin antibody was from BD Biosciences (Franklin Lakes, NJ). Snail antibody was from Abcam (Cambridge, MA), and antibodies against ERK1/2, phosphor-ERK1/2 (Thr202/Tyr204), phosphor-cofilin, phospho-GSK3α/β (Ser21/Ser9), and FAK and phosphor-Y397 FAK were from Cell Signaling Technology Inc. (Danvers, MA). Cofilin antibody was from the Cytoskeleton Inc. (Denver, CO). Cell lysates were prepared for Western blotting similarly as previously described^6^,^51^.

### Cell migration and Matrigel invasion assays

Wound healing assays and Transwell migration and invasion assays were conducted similarly as we did previously^6^. For wound healing assays, at the designated time points, the images of the wound were captured under a Zeiss Axiovert 200M microscope. The relative migration rate was calculated by following the formula: (wound width at 0 hr-wound width at n hr)/wound width at 0 hr. For Transwell assays, cells were starved for 4 hrs, harvested and re-suspended in the serum-free medium containing 0.1% bovine serum albumin before seeded onto Transwell chambers. The insert membranes in the invasion assays were coated with 0.25 μg/ml Matrigel. The experiments were repeated for three times, and the results were analyzed using Student’s *t*-test.

### Immunofluorescence, phase imaging and cell spreading assays

Immunofluorescence was conducted similarly as described previously. Focal adhesions were stained with a vinculin antibody and visualized with Alexa Fluor 594-conjugated goat anti-mouse IgG (H). Confocal images were acquired with the BD Pathway 855 imaging station. For phase imaging, cells were cultured in a 6-well plate overnight, or for 24 hrs (MCF-7), and images were taken with a Zeiss Axiovert 200M microscope. For spreading assays, cells were plated onto fibronectin coated dishes, phase contrast images were taken with a Nikon Eclipse TE2000 microscope. For quantification of focal adhesions, 25 cells each field and a total of three fields were counted using the ImageJ software (rsb.info.nih.gov/ij) and the data were analyzed using Student’s *t*-test.

### MTT cell proliferation and soft agar assays

Approximately 4,000 cells were seeded onto each well of a 96-well plate. At each time point, the cells were incubated with 15 μl Dye solution for 4 hrs at 37 °C followed by lysis of cells. The released formazan product was detected with an Epoch Microplate Spectrometer (BioTek) at a 570 nM absorbance reading. For soft agar assays, approximately 5,000 cells were mixed with 2 ml of complete medium plus 0.35% agar. The agar-cell mixture was plated on top of a bottom layer made of complete medium containing 0.5% agar. After solidification, 2 ml of liquid complete medium was added. After 4 weeks, the colonies were stained with 1.0% crystal violet and photographed under a Nikon Eclipse TE2000 microscope. Numbers of colonies in five random fields were scored. The experiment was conducted for three times, and the data were analyzed using Student’s *t*-test.

## Additional Information

**How to cite this article**: Zhang, H. and Zhou, G.-L. CAP1 (Cyclase-Associated Protein 1) Exerts Distinct Functions in the Proliferation and Metastatic Potential of Breast Cancer Cells Mediated by ERK. *Sci. Rep.* **6**, 25933; doi: 10.1038/srep25933 (2016).

## Supplementary Material

Supplementary Information
