# Supplementary material for: CAP1 (Cyclase-Associated Protein 1) Exerts Distinct Functions in the Proliferation and Metastatic Potential of Breast Cancer Cells Mediated by ERK
Source: Sci Rep. 2016 May 13;6:25933. doi: 10.1038/srep25933 (PMC4865817; doi:10.1038/srep25933)
Supplement: Supplementary Information [file srep25933-s1.pdf]

Supplementary Figures and Figure legends

CAP1 (Cyclase-Associated Protein 1) Exerts Distinct Functions in the Proliferation and Metastatic Potential of Breast Cancer Cells Mediated by ERK

Haitao Zhang[1, 2] & Guo-Lei Zhou[1, 2, #]

1. Department of Biological Sciences, Arkansas State University, State University, AR 72467, U.S.A.

2. Molecular Biosciences Program, Arkansas State University, State University, AR 72467, U.S.A.

Running title: Cell Context-Dependent Functions for CAP1 in Breast Cancer

NOTE:

#: To whom correspondence should be addressed: Guo-Lei Zhou, Department of Biological Sciences, Arkansas State University; P.O. Box 599, State University, AR 72467, U.S.A.

Email: gzhou@astate.edu; Tel: 870-680-8588. Fax: 870-680-4347

Supplementary Figure S1

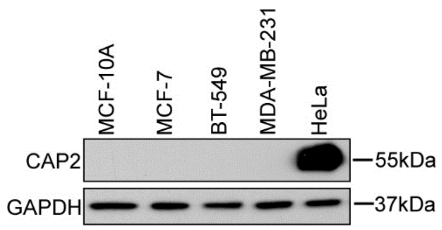

Supplementary Figure S2

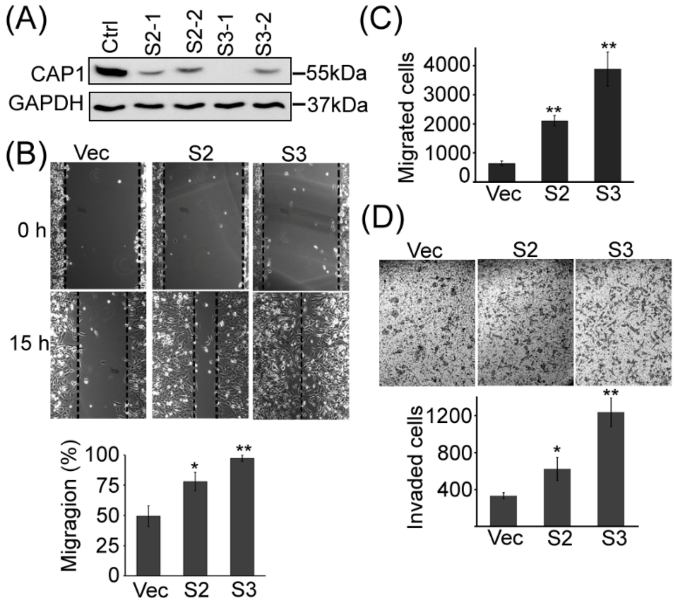

Supplementary Figure S3

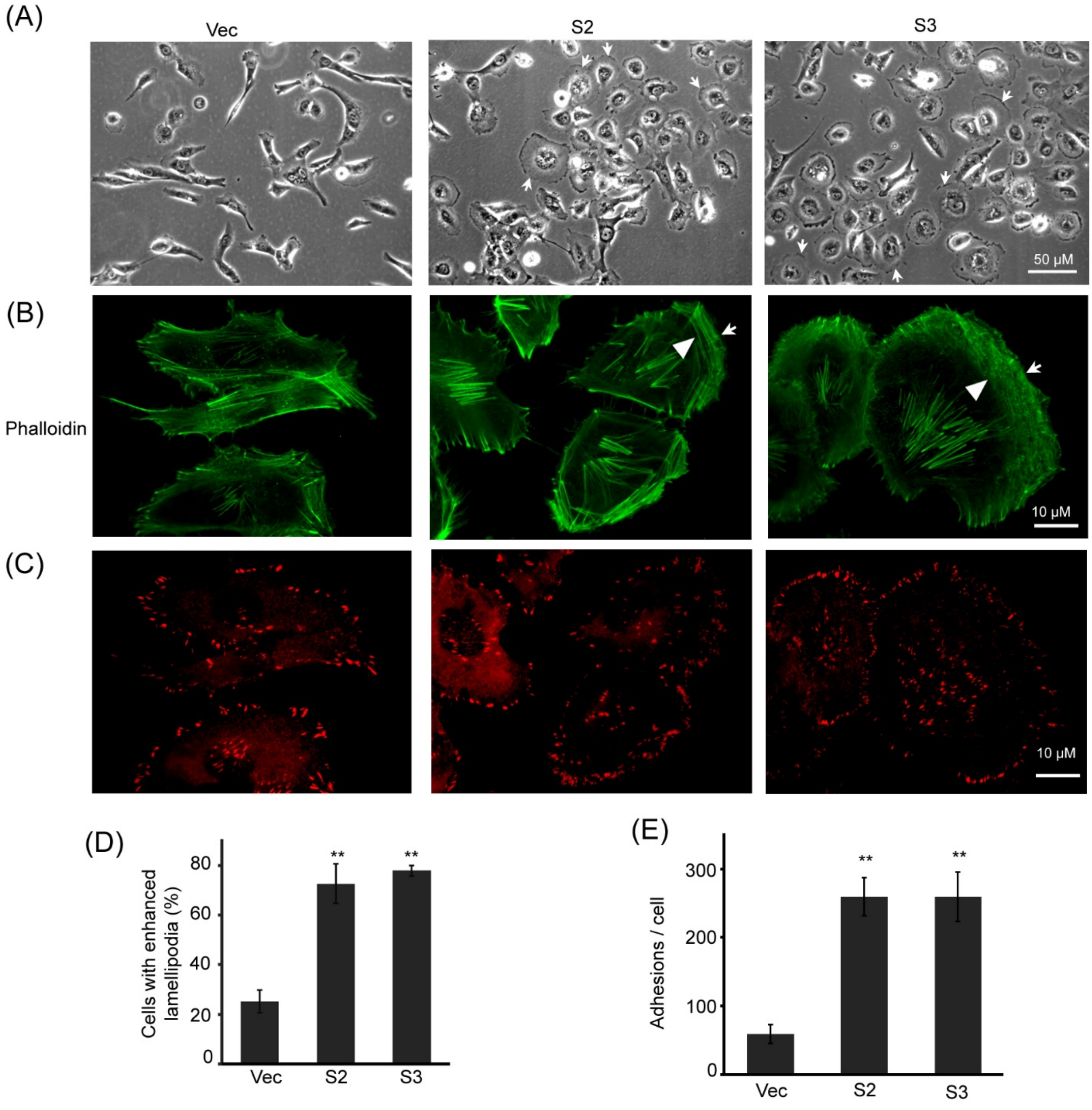

Supplementary Figure S4

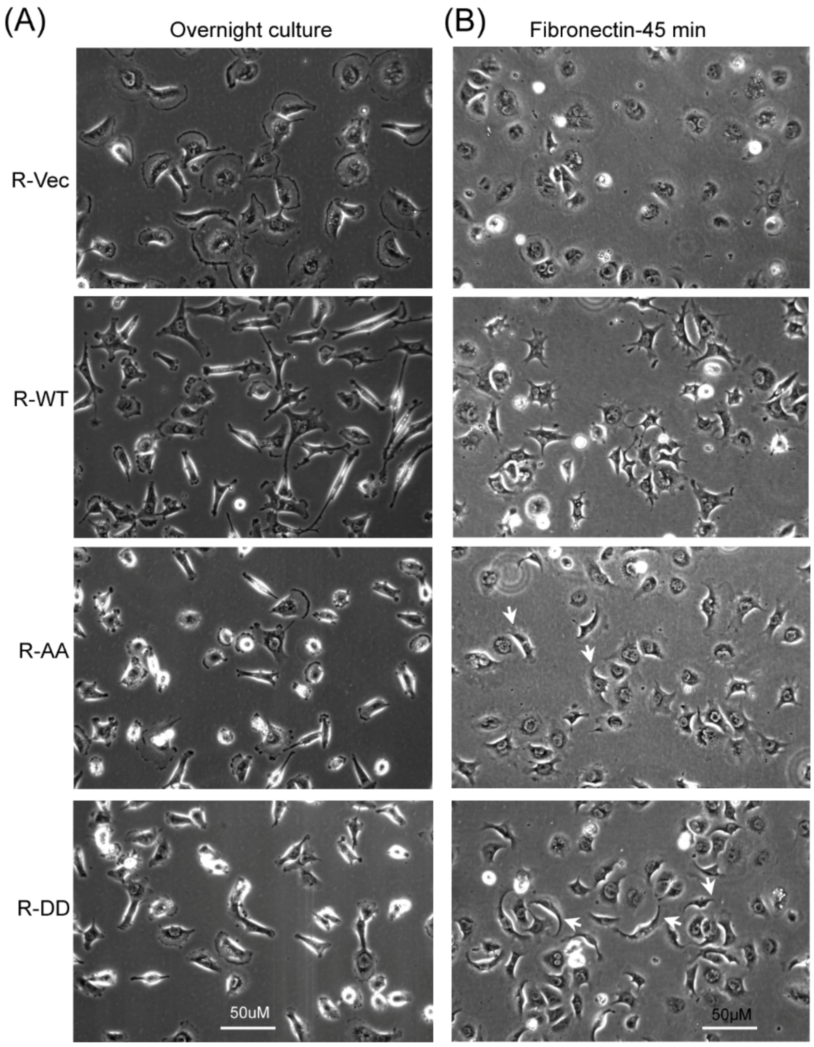

Supplementary Figure S5

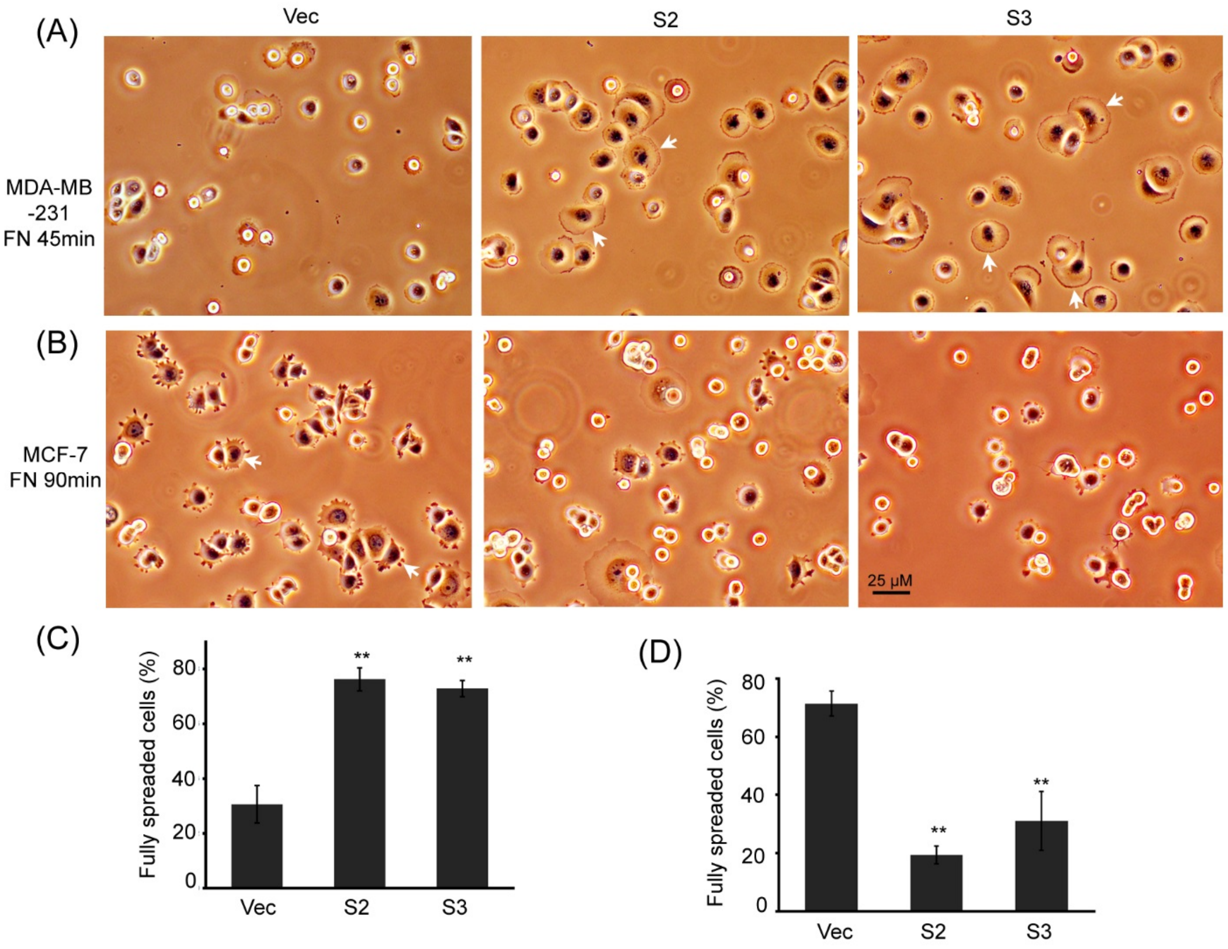

Supplementary Figure S1. Undetectable CAP2 expression in normal breast cells and breast cancer cells. Cell lysates were resolved in SDS-PAGE followed by Western blotting with a polyclonal CAP2 antibody, with GAPDH serving as a loading control. Lysate of HeLa cells, which are known to express high levels of both CAP1 and CAP2, was used as a control.

Supplementary Figure S2. Depletion of CAP1 stimulated the migration and invasion in MDA-MB-231 cells. (A) Efficient knockdown by both the S2 and S3 shRNA constructs was confirmed in Western blotting, with GAPDH serving as a loading control. Two stable clones were established and used for each shRNA construct. **(B)** Wound healing assays show that knockdown of CAP1 stimulated the migratory capability. The Y-axis of the graph represents the relative healed area with the error bar representing S.E.M. (**, $P< 0.01$; *, $P< 0.05$ vs. Vec, n=3). **(C)** Transwell migration assays show that CAP1 knockdown increased the migration of MDA-MB-231 cells. The Y-axis of the graph represents the mean of migrated cells with the error bar representing S.E.M. (**, $P< 0.01$ vs. Vec, n=3). **(D)** Matrigel invasion assays show that CAP1 knockdown increased the cell invasion. The Y-axis of the graph represents the mean number of the invaded cells with the error bar representing S.E.M. (**, $P< 0.01$; *, $P< 0.05$ vs. Vec, n=3). The upper panels are the representative field of invaded cells taken with microscopy.

Supplementary Figure S3. CAP1 knockdown promoted formation of pro-migratory subcellular structure in the metastatic BT-549 cancer cells. (A) Bright field images of control (Vec) and stable knockdown cells (S2 and S3) show the cell morphological changes associated with migration capability. The arrows indicated the broad lamellipodia in the CAP1 knockdown cells. **(B)** Confocal images show the actin cytoskeleton that was stained by Phalloidin. Arrows indicate the lamellipodia

and the arrowheads indicate the actin arcs at the leading edge. **(C)** Confocal images show the focal adhesions stained by a vinculin antibody. Arrows indicated the focal adhesions. 12 hours after seeded onto fibronectin-coated Mattek dishes, the cells were fixed and stained. **(D)** Statistic analysis of cells with enhanced protrusions in the control and CAP1 knockdowns cells in (A). The total cell number (at least 70) and number of cells with enhanced protrusions per field were counted in five random fields. The percentage of cells with enhanced protrusions was calculated, analyzed in Student's *t*-test and shown in the graph with the error bar representing S.D. (**, P< 0.01 vs. Vec, n=5). **(E)** Statistic analysis of the number of focal adhesions per cell. The numbers of focal adhesions per cell (C) were counted using the Image J program, and 25 cells per filed were counted. The experiment was repeated three times and the average numbers were represented in the graph with the error bars representing S.D. (**, P< 0.01 vs. Vec, n=3).

Supplementary Figure S4. Rescue of cell morphology by re-expression of WTCAP1 and phosphor mutants in CAP1 knockdown BT-549 cells. (A) Bright field images showing the rescue of cell morphology by WT CAP1 and the AA and DD phosphor mutants in the CAP1 knockdown BT-549 cells cultured overnight under normal conditions. **(B)** Bright field image showing the rescue of cell morphology by WT CAP1 and the AA and DD phosphor mutants in the CAP1 knockdown BT-549 cells 45 minutes after cells were plated on fibronectin coated surfaces. The arrows indicate the large but not correct-positioned lamellipodia in the cells re-expressing the AA and DD mutants.

Supplemental Figure 5. Phase contrast imaging shows distinct effects of CAP1 knockdown on the spreading of MDA-MB-231 and MCF-7 cells on fibronectin coated surface. (A) MDA-MB-231 cells were seeded onto fibronectin coated dishes, cultured for 45 minutes and phase images were

taken. Arrows indicate the broad lamellipodia in fully spread cells. **(B)** MCF7 cell were plated onto fibronectin-coated dishes, but the cells were allowed to attach for 90 minutes before the phase images were taken. **(C&D)** Statistical analysis results show distinct effects of CAP1 depletion on the spreading of the MDA-MB-231 (C) and MCF-7 (D) cells. At least 50 total cells per field were counted in three random fields and the percentages of fully spread cells were calculated. The experiment was repeated for three times and the data were analyzed in Student's *t*-test and shown in the graphs with the error bar representing S.E.M. (**, $P< 0.01$ vs. Vec, n=3). Cells without bright white circle were counted as spread cells (arbitrary definition).
